# Decomposing the gaps in healthy and unhealthy life expectancies between Indigenous and non-Indigenous Australians: a burden of disease and injury study

**DOI:** 10.1186/s12963-024-00335-z

**Published:** 2024-07-11

**Authors:** Yuejen Zhao, Renu Unnikrishnan, Ramakrishna Chondur, Jo Wright, Danielle Green

**Affiliations:** Health Statistics & Informatics Branch, Northern Territory Department of Health, Floor 6, Manunda Place, 38 Cavenagh St, Darwin, PO Box 40596, Casuarina, NT 0811 Australia

**Keywords:** Life expectancy, Decomposition, Population health, Burden of disease and injury, Healthy life expectancy

## Abstract

**Background:**

The gaps in healthy life expectancy (HLE) between Indigenous and non-Indigenous Australians are significant. Detailed and accurate information is required to develop strategies that will close these health disparities. This paper aims to quantify and compare the causes and their relative contributions to the life expectancy (LE) gaps between the Indigenous and non-Indigenous population in the Northern Territory (NT), Australia.

**Methods:**

The age-cause decomposition was used to analyse the differences in HLE and unhealthy life expectancy (ULE), where LE = HLE + ULE. The data was sourced from the burden of disease and injury study in the NT between 2014 and 2018.

**Results:**

In 2014–2018, the HLE at birth in the NT Indigenous population was estimated at 43.3 years in males and 41.4 years in females, 26.5 and 33.5 years shorter than the non-Indigenous population. This gap approximately doubled the LE gap (14.0 years in males, 16.6 years in females) at birth. In contrast to LE and HLE, ULE at birth was longer in the Indigenous than non-Indigenous population. The leading causes of the ULE gap at birth were endocrine conditions (explaining 2.9–4.4 years, 23–26%), followed by mental conditions in males and musculoskeletal conditions in females (1.92 and 1.94 years, 15% and 12% respectively), markedly different from the causes of the LE gap (cardiovascular disease, cancers and unintentional injury).

**Conclusions:**

The ULE estimates offer valuable insights into the patterns of morbidity particularly useful in terms of primary and secondary prevention.

**Supplementary Information:**

The online version contains supplementary material available at 10.1186/s12963-024-00335-z.

## Background

Life expectancy (LE) and healthy life expectancy (HLE) are some of the most important health outcome measures for assessing population health and the effectiveness of population-level interventions [1, 2]. HLE is a useful measure of population health that incorporates both the quantity and quality of life [3–6]. Quality of life has long been studied and recognised using different terms in early literature such as failure of success [7], compression of morbidity [8] and equilibrium theory of mortality and morbidity [9]. Improved LE will inevitably increase morbidity of chronic disease and demand for health services [10]. Unhealthy or ill-health LE (ULE) is the difference between LE and HLE, designed to measure the non-fatal burden of disease and injury (BOD) [11]. Substantial gaps in HLE between the Indigenous and non-Indigenous populations have been observed in Australia [11, 12]. There is also considerable geographic variation in BOD closely associated with remoteness [11]. The Northern Territory (NT) is the most remote jurisdiction in Australia with the highest proportion of Aboriginal and Torres Strait Islander (hereafter respectfully referred to as Indigenous) peoples (26% in 2021), of whom majority (almost 70%) live in remote and very remote areas [13]. Indigenous people in the NT are overrepresented among the most disadvantaged, with significantly shorter LE, poor quality of life and greater health care needs [11, 13]. 

LE is a summary measure for mortality, which can be used to evaluate the effectiveness of life-saving health interventions [14]. However, LE does not fully reflect the morbidity and disability caused by diseases and injuries. People want to live longer and healthier lives by improving both longevity and quality of life [8]. HLE provides a more comprehensive picture of population health by summarising a wide range of epidemiological measures of disease and injury including both morbidity and mortality [14]. In Australia, the LE and HLE for the Indigenous and non-Indigenous population are well documented [11, 15]. However, little is known about what types of illness constitute the ULE and what drives the difference in ULE between Indigenous and non-Indigenous Australians.

This study aims to quantify the contribution of illness to ULE and the gap in ULE by Indigenous status, in comparison with LE to shed light on the causes of the health gaps between the Indigenous and non-Indigenous population by using the age and cause decomposition techniques.

## Method

This is a cross-sectional observational study on the LE patterns for NT residents between 2014 and 2018 by key demographic variables (5-year age group, sex and Indigenous status) and type (healthy or unhealthy), using death and illness information from the fourth NT BOD study [12, 16]. The BOD study analysed 5593 deaths and 520,195 hospitalisations for 2014–2018 with routine quality control for causes of death and diagnoses. The cause of death unit record files were sourced from the Australian Coordinating Registry including all deaths registered in Australia, together with demographic information of the deceased NT residents [17]. Accuracy of the hospital demographic data has been assessed to be about 97% [18]. Because of the small population size in the NT, the annual death, hospitalisation and population estimates were combined into a 5-year period (population totalled 1,227,839 for 2014–2018). Other data sources used for years lived with disability (YLD) estimation included health survey data, emergency department and primary care data, and statutory surveillance data, such as communicable disease notifications, perinatal and cancer registry records. The level of disability in YLD estimation was measured by disability weight ranging from 0 (perfect health) to 1 (death). The BOD study followed the methodologies used in the national and global BOD studies including disability weights for consistency and comparability [12]. The disability weights were multiplied by the number of years spent living with a condition to obtain the YLD. Age-specific mortality and YLD rates were analysed by using standard BOD categories [12, 16, 19, 20], based on the International Statistical Classification of Diseases and Related Health Problems. Abridged period life table and the Sullivan method were applied to measure LE, HLE and ULE, where ULE = LE-HLE [3, 21]. HLE enhances the idea of LE by considering YLD. HLE was derived using prevalence based YLD estimates from the NT BOD study and Indigenous and non-Indigenous life table [10]. 

Further analysis of the gaps is based on age and cause decomposition using the Arriaga method for LE and the Andreev method for HLE [22, 23]. Descriptions of generalised age and cause decompositions for LE, HLE, ULE and the demographic gaps ${\Delta}$ between two populations are provided in Appendices (1 and 2).

## Results

**Fig. 1:**
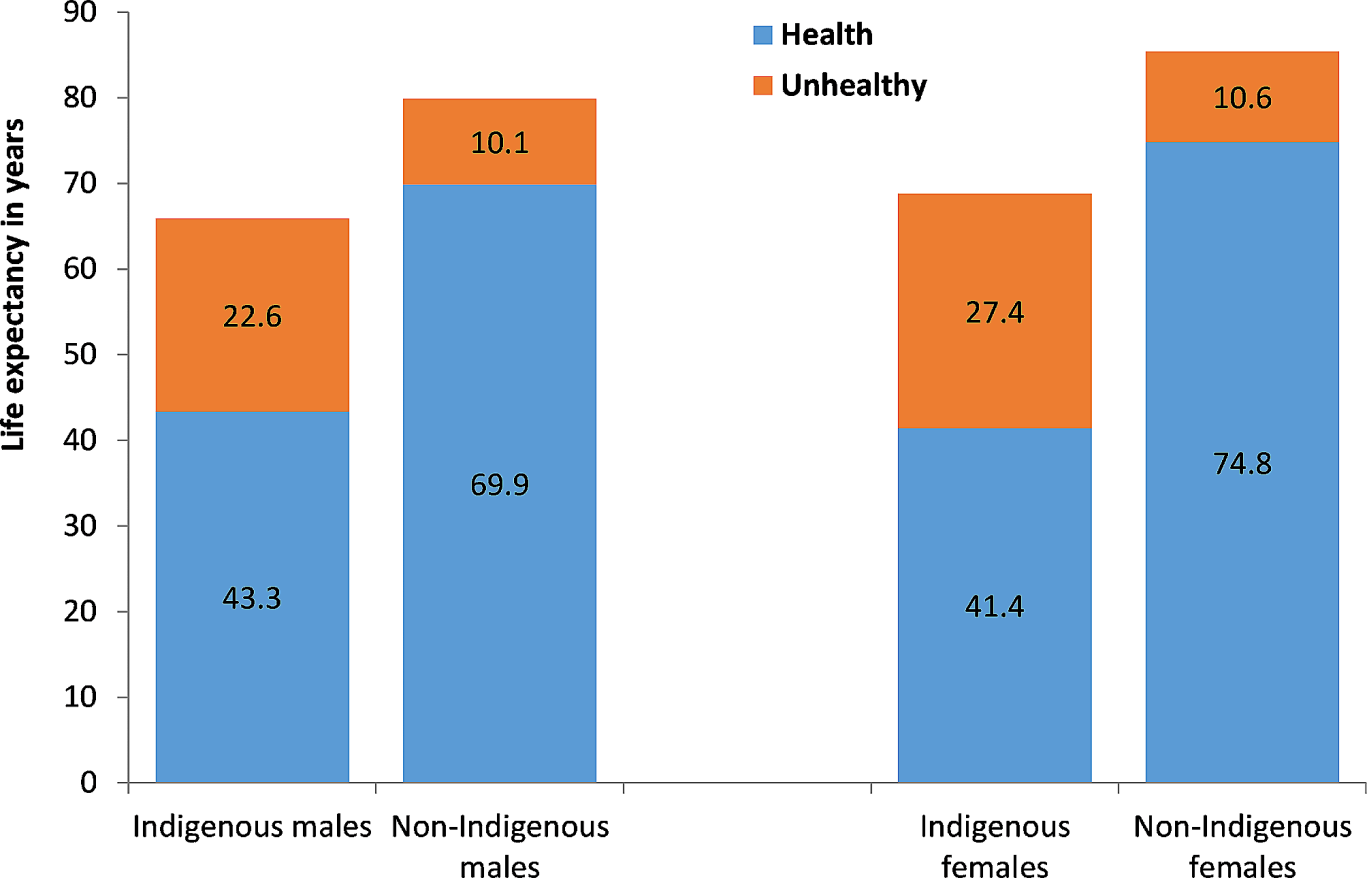
Healthy and unhealthy life expectancy at birth by sex and Indigenous status, Northern Territory, 2014–2018

In the NT between 2014 and 2018, LE at birth for Indigenous and non-Indigenous males was 65.9 and 80 years respectively, whereas LE at birth was 68.8 and 85.4 years for Indigenous and non-Indigenous females (see Fig. 1). Indigenous males at birth could be expected to live 14.1 years less than their non-Indigenous counterparts. However, Indigenous males lived 26.6 years less in full health and spent 12.5 years longer in poor health than their non-Indigenous counterparts. Indigenous females could be expected to live 16.6 years less than non-Indigenous females and 33.4 years less in full health at birth, spending 16.8 years longer in ill-health than their non-Indigenous counterparts. HLE at birth for Indigenous males and females was only 43.3 and 41.4 years. The HLE gap at birth was approximately double the size of the LE gap, indicating NT Indigenous people not only live shorter lives, but also lived for more years in poor health. ULE showed greater disparities, being 2.2 and 2.6 times longer in Indigenous males and females, respectively compared with the non-Indigenous counterparts.

It is important to further investigate which conditions were associated with longer ULE for Indigenous people. The decomposition analysis of ULE from Table 1 shows that mental / substance-use conditions made the largest contribution to ULE in Indigenous males accounting for 3.43 years, followed by endocrine conditions (3.31 year) and musculoskeletal disorders (2.87 years). Endocrine conditions contributed most to ULE in Indigenous females (4.81 years), followed by musculoskeletal disorders (4.37 years) and mental / substance-use conditions (3.09 years). The non-Indigenous population had a very different pattern for causes of ULE with the leading cause being musculoskeletal disorders (1.9 and 2.44 years in males and females), followed by mental / substance-use disorder (1.52 and 1.28 years in males and females), and then intentional injury in males (0.93 years) and respiratory disease in females (1.1 years).

**Table 1:** Cause decomposition of unhealthy life expectancy at birth (contributions %)[rank] by indigenous status and cause category, Northern Territory Australia, 2014–2018

Cause*	Indigenous			Non-Indigenous	
	Male	Female		Male	Female
Musculoskeletal	2.87(13%)[3]	4.37(16%)[2]		1.90(19%)[1]	2.44(23%)[1]
Mental/Substance	3.43(15%)[1]	3.09(11%)[3]		1.52(15%)[2]	1.28(12%)[2]
Endocrine	3.31(15%)[2]	4.81(18%)[1]		0.44(4%)[10]	0.40(4%)[9]
Cardiovascular	2.05(9%)[5]	2.33(9%)[5]		0.85(8%)[4]	0.75(7%)[5]
Respiratory	1.09(5%)[9]	2.49(9%)[4]		0.74(7%)[5]	1.10(10%)[3]
Neurological	1.35(6%)[8]	1.62(6%)[8]		0.70(7%)[6]	1.02(10%)[4]
Intentional inj.	2.15(10%)[4]	1.26(5%)[9]		0.93(9%)[3]	0.47(4%)[7]
Hearing/Vision	1.92(9%)[6]	1.91(7%)[7]		0.34(3%)[11]	0.49(5%)[6]
Kidney/Urinary	1.44(6%)[7]	2.11(8%)[6]		0.52(5%)[7]	0.31(3%)[11]
Unintentional inj.	1.03(5%)[10]	0.53(2%)[12]		0.48(5%)[9]	0.27(3%)[14]
Cancer	0.17(1%)[14]	0.19(1%)[16]		0.52(5%)[8]	0.47(4%)[8]
Infectious	0.45(2%)[12]	0.55(2%)[10]		0.26(3%)[13]	0.27(3%)[13]
Skin	0.36(2%)[13]	0.50(2%)[14]		0.27(3%)[12]	0.29(3%)[12]
Oral	0.51(2%)[11]	0.51(2%)[13]		0.20(2%)[15]	0.21(2%)[16]
Gastrointestinal	0.16(1%)[15]	0.19(1%)[17]		0.21(2%)[14]	0.25(2%)[15]
Blood/Metabolic	0.15(1%)[16]	0.55(2%)[11]		0.06(1%)[17]	0.18(2%)[17]
Reprod./Maternal	0.01(0%)[18]	0.33(1%)[15]		0.01(0%)[18]	0.33(3%)[10]
Infant/Congenital	0.12(1%)[17]	0.09(0%)[18]		0.09(1%)[16]	0.07(1%)[18]
	22.58(100%)	27.43(100%)		10.05(100%)	10.59(100%)

* Ranked in descending order by contributions to the total unhealthy life expectancy; inj = injury; Reprod = reproductive

The differences in the causes of ULE between the Indigenous and non-Indigenous population are shown in Table 2 using age decomposition of the gaps (Appendix 2). The condition causing the most difference was endocrine conditions for both males and females, explaining 23% and 26% of the total differences respectively, followed by mental / substance-use and hearing / vision conditions (contributing 15% and 13% respectively) in males and musculoskeletal and mental / substance-use conditions (12% and 11%) in females.

**Table 2:** Cause decomposition of the gap in unhealthy life expectancy at birth between Indigenous and non-Indigenous population by sex, Northern Territory Australia, 2014–2018

Cause*	Male		Cause*	Female
Endocrine	2.87(23%)[1]		Endocrine	4.41(26%)[1]
Mental/Substance	1.92(15%)[2]		Musculoskeletal	1.94(12%)[2]
Hearing/Vision	1.58(13%)[3]		Mental/Substance	1.81(11%)[3]
Intentional inj.	1.21(10%)[4]		Kidney/Urinary	1.80(11%)[4]
Cardiovascular	1.19(10%)[5]		Cardiovascular	1.58(9%)[5]
Musculoskeletal	0.97(8%)[6]		Hearing/Vision	1.41(8%)[6]
Kidney/Urinary	0.92(7%)[7]		Respiratory	1.39(8%)[7]
Neurological	0.65(5%)[8]		Intentional inj.	0.78(5%)[8]
Unintentional inj.	0.55(4%)[9]		Neurological	0.60(4%)[9]
Respiratory	0.36(3%)[10]		Blood/Metabolic	0.37(2%)[10]
Oral	0.31(2%)[11]		Oral	0.30(2%)[11]
Infectious	0.19(2%)[12]		Infectious	0.28(2%)[12]
Blood/Metabolic	0.09(1%)[14]		Unintentional inj.	0.27(2%)[13]
Skin	0.09(1%)[13]		Skin	0.21(1%)[14]
Infant/Congenital	0.03(0%)[15]		Infant/Congenital	0.02(0%)[15]
Reprod/Maternal	0.00(0%)[16]		Reprod/Maternal	0.00(0%)[16]
Gastrointestinal	-0.05(0%)[17]		Gastrointestinal	-0.06(0%)[17]
Cancer	-0.35(-3%)[18]		Cancer	-0.28(-2%)[18]
Total	12.53(100%)		Total	16.84(100%)

* Ranked in descending order by contributions to the gap (years); inj = injury; Reprod = reproductive

**Fig. 2:**
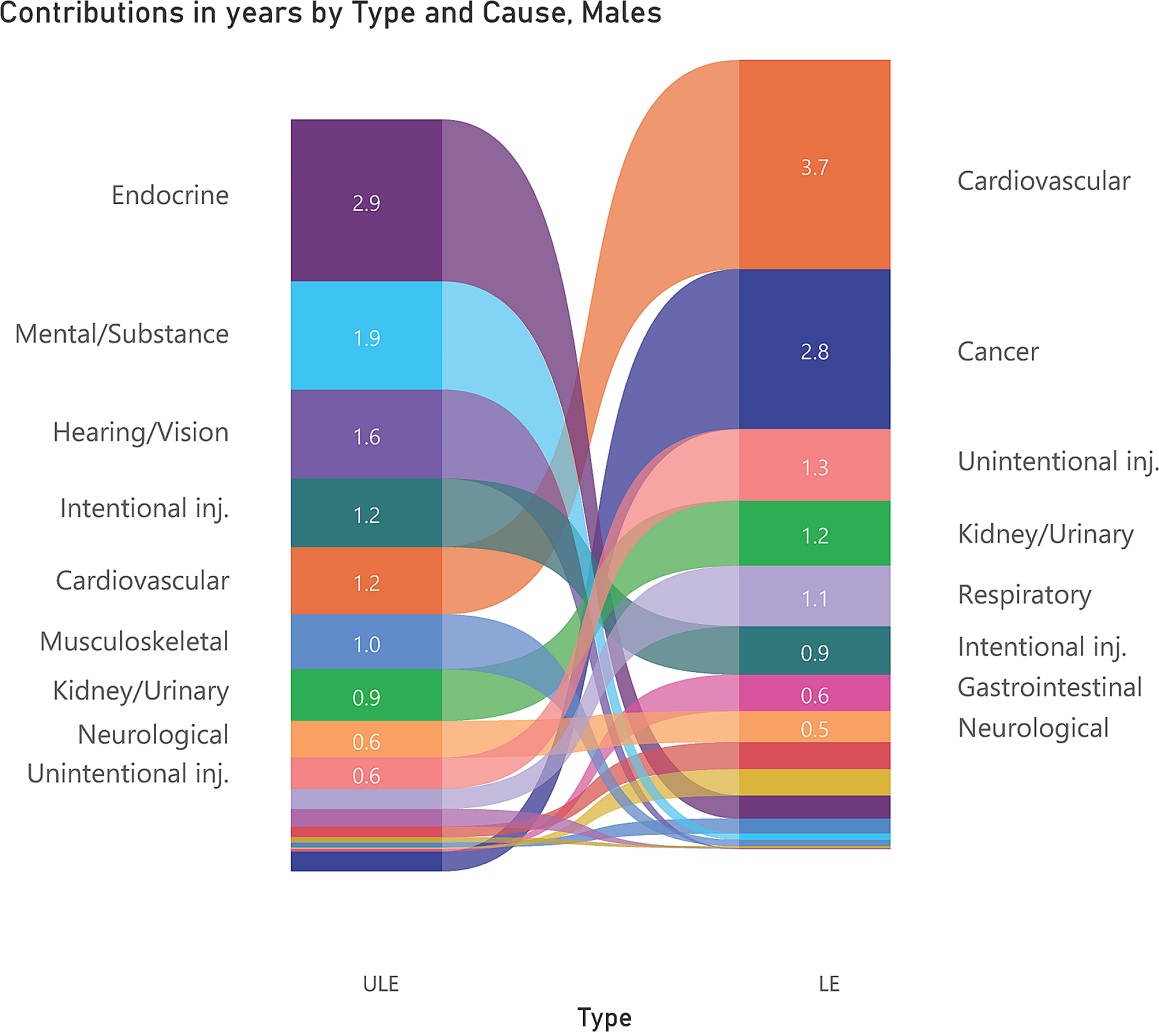
Comparison of cause decompositions for Indigenous life expectancy (LE) and unhealthy life expectancy (ULE) gaps at birth in years, males, Northern Territory Australia, 2014–2018

**Fig. 3:**
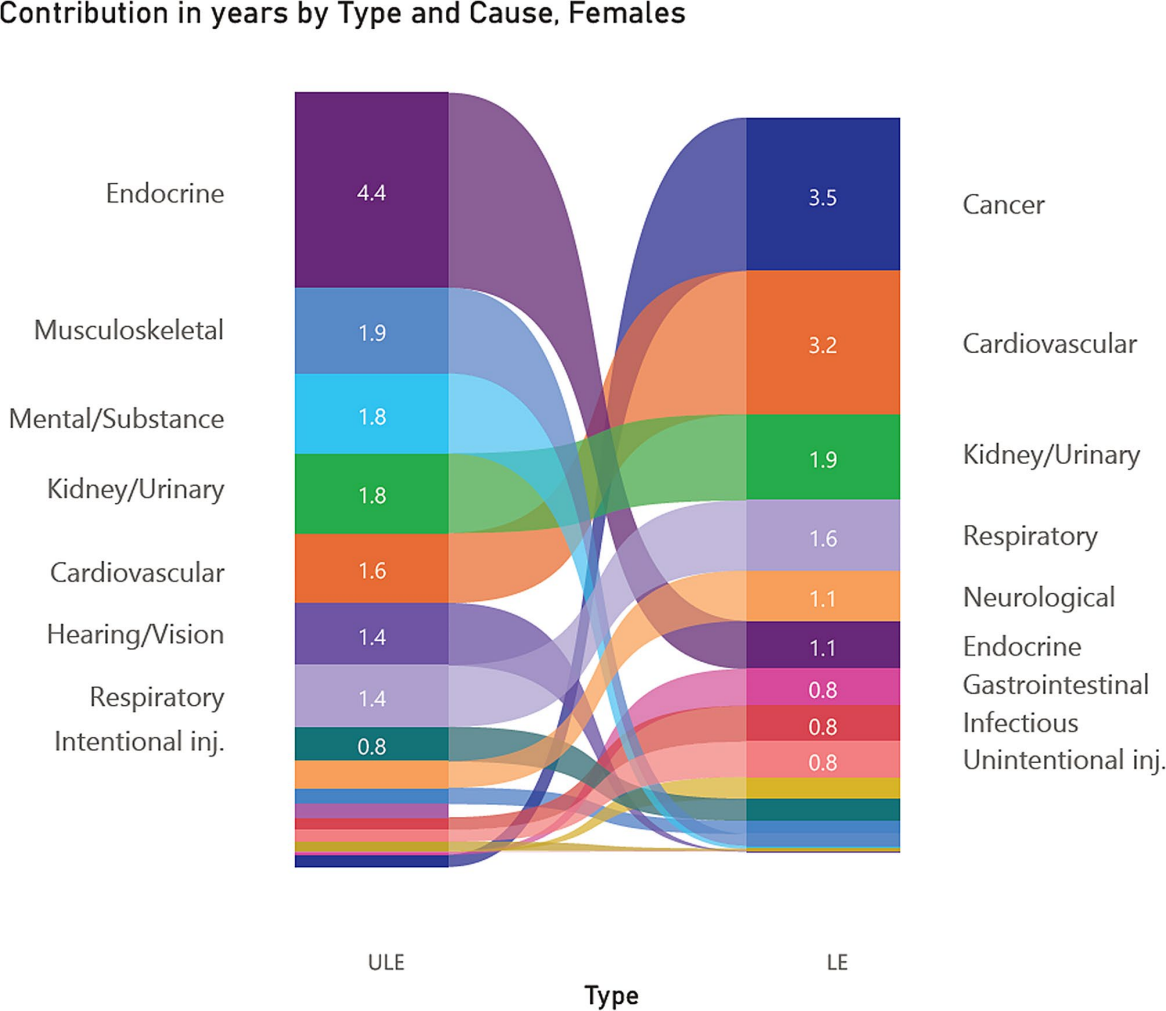
Comparison of cause decompositions for Indigenous life expectancy (LE) and unhealthy life expectancy (ULE) gaps at birth in years, females, Northern Territory Australia, 2014–2018

Figures 2 and 3 show the different causes of ULE and total LE. It is evident the leading causes of the gap between the Indigenous and non-Indigenous population in ULE were very different from the leading causes for LE.

Figure 2 indicates that the top three conditions causing the Indigenous LE gap in males were cardiovascular (contributing 3.7 years), cancer (2.8 years) and unintentional injury (1.3 years), whereas the top three conditions causing the Indigenous ULE gap in the same male population were endocrine (contributing 2.9 years), mental / substance-use (1.9 years) and hearing / vision conditions (1.6 years). Figure 3 shows that for females the top ranked three conditions causing the Indigenous LE gap were cancer (contributing 3.5 years), cardiovascular (3.2 years) and kidney diseases (1.9 years), whereas the top three conditions causing the Indigenous ULE gap in the same female population were endocrine (contributing 4.4 years), musculoskeletal (1.9 years) and mental / substance-use conditions (1.8 years).

HLE is the balance between LE and ULE, and the impact of the causes on HLE is more complex. This study shows that the gap in HLE between the Indigenous and non-Indigenous population was negatively impacted by a combination of the drivers of both LE and ULE including endocrine disorders (7.9–10.4 years, 30–31%), cardiovascular diseases (4.7–7.1 years, 18–21%) and hearing / vision conditions (5.2–6.6 years, 16–25%). More details on YLD are provided in Appendix 3 (Table A1). Information on cause decomposition of Indigenous HLE gap is provided in Appendix 4 (Table A2).

## Discussion

Between 2014 and 2018, LE at birth for Indigenous males and females was 65.9 and 68.8 years, 14.1 and 16.6 years respectively behind the non-Indigenous population in the NT. The NT Indigenous LE gaps were about 3.9–7.5 years wider than the Indigenous LE gaps nationally (10.2 years for male and 9.1 years for females) [11]. However, the NT HLE for Indigenous males and females was only 43.3 and 41.4 years, 26.6 and 33.4 years behind the NT non-Indigenous counterparts, almost doubling the national Indigenous HLE gaps (15.1 years in males and 13.9 years in females) [11, 12]. Consequently, the NT Indigenous males and females had 12.5 and 16.8 years longer ULE than the non-Indigenous counterparts, approximately 2.6–3.7 times greater than the national gap [11], contributing to the higher demand for health care services. To put this into context, HLE in the NT Indigenous population was low according to international comparisons (the NT Indigenous population was equivalent to poor underdeveloped countries) [1]. On average, Indigenous males and females could only expect to live 65.7% and 60.2% of their lives in full health. This reflects the high BOD in the NT and high proportion of Indigenous population especially in the remote and very remote areas, and high levels of health inequity in the NT compared to other States and Territories in Australia [24–26]. 

In common with the national results [11], the gap in HLE between Indigenous and non-Indigenous people was greater than the gap in LE, indicating that HLE is a more sensitive measure for health inequality. Both an increase in LE and a decrease in ULE can lead to a longer HLE. The HLE at birth for the Indigenous population was substantially lower than the HLE for the non-Indigenous population, showing a greater level of disparity than that observed with LE, whereas ULE at birth was longer in the Indigenous population compared with the non-Indigenous population. Indigenous people had a shorter LE and HLE, but a longer ULE. In line with previous research [27], females live longer LE than males in both Indigenous and non-Indigenous population. However, female HLE fell behind male HLE in the NT Indigenous population. The Indigenous females had almost two years less HLE than their male counterparts. The reason for this disparity was that NT Indigenous females experienced 20% higher YLD than the NT Indigenous males on a per-capita basis [12]. This is not the case nationally [11]. 

Analysis of the LE, HLE and ULE gaps between the Indigenous and non-Indigenous population by causes is useful to better understand the reasons for these gaps and help those in policy and health service provision identify health priorities to reduce and eliminate the gaps. LE, HLE and ULE provide insights into different aspects of population health: The focus of LE is on longevity mainly determined by fatal conditions, ULE represents health service and support needs and reflects the level of disability, while HLE is designed to address the ultimate goal of health improvement, to minimise human suffering. Our study demonstrated the usefulness of ULE in reflecting health service need.

To the best of our knowledge, this study is the first study on ULE gaps between Indigenous and non-Indigenous population achieved by using routine BOD data. Contrary to other LE and HLE studies, this study revealed the NT female HLE was much shorter than the male HLE, indicating further investigation is required on the quality of life of Indigenous females in the NT. Our study showed endocrine disorders (mainly type 2 diabetes) contributed the most to the Indigenous ULE gap, followed by musculoskeletal and mental / substance-use conditions. This result is consistent with previous studies and will contribute to existing literature [28–33]. The dominance of endocrine disorder in Indigenous ULE gap points to the needs to strengthen coordinated health care including nutrition, education and health promotion programs for diabetes and metabolic syndrome in Australian Indigenous remote communities [34]. This study provides a more general approach to LE, HLE and ULE gap decomposition, which will allow us to gain insights about population health (morbidity and mortality) for each underlying cause [4]. This study has improved the existing age and cause decomposition method to deal with age specific mortality and morbidity effects consistently across LE, HLE and ULE [23, 35]. This study showed that, in terms of the difference between the Indigenous and non-Indigenous population, most life years were lost due to cancers and unintentional injuries and over half of the higher level of disability was caused by endocrine conditions in the Indigenous population. ULE is much more accurate and sensitive than LE and HLE in reflecting morbidity, because ULE is a more direct reflection of prevalence and health care need, and can be directly analysed by causes.

A few limitations should be noted for this study. First, our abridged period life table construction was based on the retrospective observational BOD study. The data collections were subject to incompleteness and inaccuracy. However, Australian Bureau of Statistics assessed that the NT had the lowest Indigenous under-identifications in mortality and population for LE estimations [36]. Second, there was no consideration of comorbidities and interactions between different causes in the BOD study. The prevalence of the disease and level of disability were assessed independently for each condition. Under such a scenario, there was theoretically a possibility that the total disability weight could exceed unity for an individual who suffered from severe and multiple co-morbid health conditions. This study used the Sullivan method for HLE [21], which does not consider the dynamics and interactions of different conditions. Third, the HLE and ULE gaps between the Indigenous and non-Indigenous population in Australia are substantial and underpinned by the enormous health challenges of chronic diseases including diabetes, ischemic heart disease and cancers. The root causes of the Indigenous LE, HLE and ULE gaps are complicated and include socioeconomic determinants such as living condition, education, employment, the impact of colonisation and a lack of primary care to name a few [28, 37, 38], which cannot be rectified by improving health services alone.

The cause decomposition method offers a more accurate approach to analysing life expectancies, which provides better information for improving health service delivery, planning and population health. LE, HLE and ULE are useful to show different aspects of population health by focusing on longevity, quality of life and health demand respectively. It is essential to know what drives life expectancies in order to reduce health disparities.

### Electronic supplementary material

Below is the link to the electronic supplementary material.

Supplementary Material 1

## Data Availability

No datasets were generated or analysed during the current study.

## Abbreviations

BOD: Burden of disease and injury
HLE: Healthy life expectancy
LE: Life expectancy
NT: The Northern Territory, Australia
ULE: Unhealthy life expectancy
YLD: Years lived with disability
