# Supplementary material for: Decomposing the gaps in healthy and unhealthy life expectancies between Indigenous and non-Indigenous Australians: a burden of disease and injury study
Source: Popul Health Metr. 2024 Jul 11;22:15. doi: 10.1186/s12963-024-00335-z (PMC11241960; doi:10.1186/s12963-024-00335-z)
Supplement: Supplementary file 1 — Supplementary Material 1 [file 12963_2024_335_MOESM1_ESM.docx]

**Appendix**

**(1) Age and cause decomposition of ULE at birth**

We define that LE*_i_* is the life expectancy at age *i*, where *i*=0, 5, 10, …85+. HLE*_i_* is the healthy life expectancy at age *i.* ULE*_i_* is the unhealthy life expectancy at age *i*, where ULE*_i_*= LE*_i_*-HLE*_i ._*

$l_{i}$ is the number of persons left alive at age *i* in the hypothetical cohort. *n* is the age interval in years (5). Following Arriaga [[1](#_ENREF_1)] and Andreev [[2](#_ENREF_2)], age decomposition of ULE at birth can be implemented using

$$\text{ULE}_{0}=\sum_{i=0}^{\infty} \text{ULE}_{0i}=\sum_{i=0}^{\infty} \left( l_{i}\text{ULE}_{i}-l_{i+n}\text{ULE}_{i+n} \right)/l_{0.}$$

Cause decomposition of ULE at birth by age *i* and cause *j* (*j*=1, 2, 3, …)

$$\text{ULE}_{0i}=\sum_{j=1}^{\infty} \text{ULE}_{0ij}=\sum_{j=1}^{\infty} \text{ULE}_{0i}{\cdot{}_{n}R}_{i}^{j}$$

where ${{}_{n}R}_{i}^{j}$= Proportion of cause *j* in total ${{}_{n}\text{YLD}}_{i}$ between ages *i* and *i+n*.

**(2) Gap decomposition of LE at birth**

$\text{ULE}_{0ik}$ is the age decomposed ULE for subpopulation *k* (*k=*1, 2), corresponding to the Indigenous and non-Indigenous population respectively. More generally, let $\text{LE=}\left\{ \text{LE, }\text{HLE}\text{, ULE} \right\}$. The gap in **LE** at birth between subpopulations can be decomposed by age and then cause:[[1](#_ENREF_1),[2](#_ENREF_2)]

$$\text{LE}_{0}=\sum_{i=0}^{\infty} \text{LE}_{0i}=\sum_{i=0}^{\infty} \left( l_{i}\text{LE}_{i}-l_{i+n}\text{LE}_{i+n} \right)/l_{0 ,}$$

$${\text{∆}\text{LE}}_{0i}=\text{LE}_{0i1}-\text{LE}_{0i2}.$$

**(3) Key data**

**Table A1. Years lived with disability by age, sex, Indigenous status and cause categories, Northern Territory of Australia, 2014-2018**

| Cause category \ Age (years) | 0- | 5- | 10- | 15- | 20- | 25- | 30- | 35- | 40- | 45- | 50- | 55- | 60- | 65- | 70- | 75- | 80- | 85+ | Total |
| --- | --- | --- | --- | --- | --- | --- | --- | --- | --- | --- | --- | --- | --- | --- | --- | --- | --- | --- | --- |
| Aboriginal Male |  |  |  |  |  |  |  |  |  |  |  |  |  |  |  |  |  |  |  |
| Infectious | 386.4 | 138.2 | 89.0 | 32.4 | 32.7 | 46.3 | 95.2 | 43.5 | 54.6 | 55.3 | 65.6 | 41.3 | 64.0 | 20.8 | 12.2 | 6.8 | 3.4 | 3.8 | 1191.4 |
| Infant | 167.2 | 26.5 | 18.8 | 24.2 | 31.3 | 24.7 | 27.8 | 19.0 | 12.5 | 9.5 | 7.7 | 5.5 | 3.6 | 2.0 | 0.8 | 0.4 | 0.2 | 0.2 | 381.8 |
| Cancer | 1.8 | 5.2 | 2.5 | 2.8 | 5.7 | 3.2 | 7.5 | 12.2 | 11.4 | 26.2 | 34.4 | 43.2 | 35.2 | 19.2 | 20.2 | 9.6 | 2.5 | 2.1 | 245.1 |
| Endocrine | 2.9 | 1.4 | 11.2 | 24.9 | 53.2 | 118.1 | 205.8 | 357.5 | 519.5 | 672.1 | 764.0 | 728.6 | 594.8 | 409.9 | 270.3 | 157.9 | 67.6 | 41.5 | 5001.3 |
| Cardiovascular | 17.5 | 214.7 | 172.8 | 82.8 | 69.5 | 78.4 | 110.5 | 170.7 | 279.7 | 393.1 | 440.0 | 428.7 | 349.8 | 257.8 | 173.7 | 56.0 | 54.9 | 26.8 | 3377.4 |
| Mental | 80.6 | 361.5 | 451.5 | 565.2 | 937.4 | 1712.1 | 1631.4 | 1610.3 | 1443.1 | 757.6 | 568.7 | 106.6 | 46.3 | 26.9 | 15.8 | 4.8 | 1.9 | 0.8 | 10322.4 |
| Neurological | 34.0 | 79.5 | 110.8 | 98.3 | 111.7 | 104.3 | 107.0 | 105.2 | 106.9 | 126.0 | 95.2 | 93.2 | 81.9 | 79.8 | 151.4 | 60.6 | 100.3 | 72.9 | 1718.8 |
| Hearing | 83.4 | 363.0 | 352.8 | 315.2 | 319.6 | 365.8 | 322.1 | 330.5 | 313.4 | 429.4 | 333.4 | 332.7 | 237.3 | 133.0 | 80.4 | 38.1 | 25.5 | 14.4 | 4390.0 |
| Respiratory | 53.6 | 122.2 | 135.3 | 118.2 | 138.2 | 183.0 | 228.8 | 200.7 | 104.5 | 151.8 | 172.0 | 95.7 | 222.9 | 140.1 | 66.8 | 34.0 | 11.7 | 8.9 | 2188.2 |
| Gastrointestinal | 2.9 | 6.9 | 7.7 | 14.6 | 24.6 | 26.9 | 30.4 | 32.8 | 40.3 | 41.8 | 40.9 | 31.3 | 21.3 | 11.8 | 8.6 | 3.4 | 1.3 | 0.9 | 348.5 |
| Kidney | 0.1 | 0.0 | 0.0 | 18.1 | 29.8 | 73.6 | 111.0 | 198.5 | 349.0 | 352.1 | 387.0 | 363.0 | 244.7 | 140.0 | 141.6 | 34.9 | 24.9 | 3.4 | 2471.8 |
| Reproductive | 0.5 | 0.3 | 0.4 | 0.7 | 2.4 | 3.7 | 5.0 | 4.6 | 3.6 | 4.3 | 3.2 | 2.7 | 1.5 | 0.6 | 0.5 | 0.1 | 0.1 | 0.1 | 34.1 |
| Skin | 196.7 | 148.0 | 155.7 | 174.5 | 69.2 | 55.9 | 65.3 | 60.0 | 45.4 | 37.7 | 30.1 | 22.0 | 27.6 | 8.5 | 8.8 | 2.4 | 1.2 | 1.4 | 1110.3 |
| Musculoskeletal | 24.7 | 81.5 | 148.5 | 118.9 | 157.6 | 421.7 | 528.1 | 657.6 | 612.5 | 760.3 | 724.6 | 638.8 | 523.7 | 229.0 | 123.4 | 63.9 | 21.0 | 14.0 | 5849.5 |
| Oral | 78.6 | 184.1 | 168.2 | 111.0 | 103.1 | 122.7 | 109.3 | 96.9 | 85.1 | 79.0 | 61.1 | 58.1 | 41.6 | 23.9 | 15.1 | 9.3 | 5.3 | 3.8 | 1356.2 |
| Blood | 315.9 | 4.6 | 8.6 | 6.7 | 6.0 | 6.6 | 6.1 | 7.0 | 6.6 | 7.3 | 13.9 | 11.4 | 8.6 | 5.0 | 3.7 | 3.0 | 1.3 | 3.3 | 425.6 |
| Intentional inj | 165.7 | 126.4 | 208.1 | 491.9 | 652.6 | 597.7 | 604.0 | 603.6 | 588.8 | 579.2 | 505.6 | 406.3 | 254.5 | 28.5 | 19.6 | 0.0 | 0.0 | 0.0 | 5832.5 |
| Unintentional inj | 114.9 | 110.0 | 169.1 | 270.3 | 307.6 | 239.8 | 242.0 | 322.9 | 272.8 | 146.3 | 143.0 | 242.6 | 158.7 | 22.8 | 16.1 | 0.8 | 0.5 | 0.1 | 2780.2 |
| Total | 1727.1 | 1974.0 | 2211.1 | 2470.7 | 3052.2 | 4184.6 | 4437.2 | 4833.6 | 4849.7 | 4629.0 | 4390.5 | 3651.6 | 2917.9 | 1559.5 | 1128.9 | 486.1 | 323.4 | 198.3 | 49025.4 |
| Aboriginal Female | 0 | 5 | 10 | 15 | 20 | 25 | 30 | 35 | 40 | 45 | 50 | 55 | 60 | 65 | 70 | 75 | 80 | 85 |  |
| Infectious | 466.1 | 122.0 | 77.0 | 55.5 | 62.0 | 55.7 | 66.5 | 57.3 | 108.9 | 59.2 | 53.9 | 60.1 | 55.2 | 37.7 | 23.5 | 13.9 | 14.1 | 7.4 | 1396.0 |
| Infant | 141.4 | 16.4 | 19.2 | 12.8 | 15.8 | 11.7 | 13.6 | 9.6 | 8.2 | 7.6 | 5.8 | 4.0 | 2.9 | 1.4 | 0.8 | 0.4 | 0.3 | 0.3 | 272.1 |
| Cancer | 1.7 | 1.8 | 1.6 | 3.3 | 3.3 | 12.0 | 9.9 | 17.5 | 27.8 | 42.6 | 34.2 | 36.7 | 49.1 | 32.6 | 18.2 | 12.7 | 7.0 | 2.8 | 314.7 |
| Endocrine | 8.4 | 3.2 | 13.7 | 62.5 | 113.8 | 212.7 | 351.5 | 545.0 | 785.5 | 1037.9 | 1123.3 | 1109.5 | 1004.3 | 695.9 | 436.2 | 254.5 | 169.2 | 103.5 | 8030.5 |
| Cardiovascular | 66.2 | 349.1 | 311.7 | 322.7 | 318.9 | 255.0 | 269.7 | 281.3 | 377.3 | 425.6 | 445.0 | 428.2 | 314.3 | 247.9 | 191.3 | 88.4 | 58.0 | 42.1 | 4792.6 |
| Mental | 22.4 | 139.2 | 204.6 | 403.7 | 893.7 | 1154.4 | 1403.0 | 1119.6 | 1014.2 | 922.2 | 622.8 | 271.6 | 187.3 | 43.5 | 44.6 | 7.8 | 7.0 | 0.4 | 8462.1 |
| Neurological | 72.6 | 104.5 | 114.3 | 101.7 | 110.9 | 125.3 | 165.2 | 170.6 | 198.9 | 201.7 | 173.0 | 169.6 | 129.9 | 128.2 | 87.8 | 104.1 | 151.6 | 159.5 | 2469.3 |
| Hearing | 63.2 | 333.7 | 308.0 | 286.6 | 289.7 | 288.1 | 301.8 | 270.7 | 315.8 | 316.4 | 373.5 | 300.9 | 283.2 | 175.5 | 109.2 | 62.2 | 52.4 | 28.6 | 4159.6 |
| Respiratory | 30.8 | 120.0 | 140.2 | 103.3 | 256.2 | 332.5 | 269.5 | 311.5 | 492.2 | 646.6 | 550.3 | 559.9 | 561.7 | 262.2 | 145.0 | 92.7 | 46.6 | 29.5 | 4950.8 |
| Gastrointestinal | 5.3 | 6.3 | 5.8 | 16.2 | 28.6 | 33.2 | 35.6 | 37.6 | 42.7 | 47.8 | 43.6 | 37.9 | 30.1 | 20.7 | 10.0 | 4.9 | 4.1 | 1.3 | 411.7 |
| Kidney | 0.1 | 0.1 | 0.1 | 44.9 | 75.5 | 154.9 | 235.7 | 368.3 | 460.2 | 561.6 | 576.8 | 591.3 | 418.8 | 281.7 | 162.7 | 70.6 | 35.9 | 0.0 | 4039.1 |
| Reproductive | 1.4 | 0.9 | 18.2 | 52.1 | 95.3 | 225.5 | 70.4 | 131.4 | 60.0 | 57.6 | 39.6 | 13.8 | 19.1 | 10.2 | 29.2 | 10.0 | 0.4 | 0.2 | 835.3 |
| Skin | 248.3 | 149.5 | 169.8 | 171.5 | 82.7 | 52.0 | 71.3 | 62.0 | 68.6 | 89.1 | 68.0 | 55.4 | 42.5 | 25.9 | 9.5 | 3.8 | 2.6 | 3.8 | 1376.3 |
| Musculoskeletal | 22.1 | 60.1 | 129.3 | 178.5 | 250.6 | 423.2 | 608.8 | 874.8 | 989.3 | 1143.2 | 1137.3 | 963.9 | 911.2 | 621.7 | 234.0 | 120.7 | 52.6 | 26.8 | 8748.1 |
| Oral | 79.2 | 168.7 | 139.4 | 94.8 | 87.4 | 107.1 | 104.0 | 89.3 | 77.3 | 76.9 | 64.5 | 64.0 | 48.5 | 30.1 | 19.3 | 15.7 | 11.1 | 6.5 | 1283.8 |
| Blood | 442.1 | 16.7 | 37.9 | 139.0 | 88.3 | 60.8 | 48.7 | 93.9 | 150.7 | 115.7 | 74.2 | 74.9 | 48.2 | 32.0 | 9.0 | 7.5 | 4.2 | 3.6 | 1447.4 |
| Intentional inj | 95.5 | 68.9 | 115.4 | 271.7 | 383.1 | 447.5 | 494.2 | 550.9 | 448.8 | 235.8 | 160.7 | 73.7 | 50.9 | 43.5 | 31.6 | 3.0 | 0.0 | 0.0 | 3475.5 |
| Unintentional inj | 60.0 | 65.8 | 77.7 | 68.1 | 89.8 | 218.1 | 237.5 | 135.0 | 123.9 | 128.6 | 99.8 | 50.7 | 35.1 | 26.4 | 18.3 | 1.0 | 0.6 | 0.3 | 1436.9 |
| Total | 1826.7 | 1727.0 | 1883.9 | 2388.7 | 3245.4 | 4169.8 | 4757.0 | 5126.3 | 5750.4 | 6116.1 | 5646.3 | 4866.2 | 4192.4 | 2717.1 | 1580.3 | 873.7 | 617.7 | 416.6 | 57901.7 |
| Non-Aboriginal Male | 0 | 5 | 10 | 15 | 20 | 25 | 30 | 35 | 40 | 45 | 50 | 55 | 60 | 65 | 70 | 75 | 80 | 85 |  |
| Infectious | 213.7 | 36.7 | 36.5 | 25.4 | 62.4 | 142.3 | 174.4 | 60.4 | 85.4 | 80.4 | 83.7 | 55.2 | 80.1 | 37.6 | 25.0 | 23.1 | 17.0 | 24.7 | 1264.0 |
| Infant | 162.2 | 24.7 | 22.7 | 23.0 | 33.7 | 48.4 | 45.2 | 36.4 | 32.9 | 24.2 | 21.7 | 19.3 | 15.6 | 11.0 | 6.4 | 3.6 | 1.6 | 1.4 | 533.9 |
| Cancer | 8.1 | 3.8 | 5.5 | 2.2 | 15.4 | 14.2 | 19.7 | 57.2 | 72.4 | 100.5 | 154.5 | 249.0 | 275.8 | 378.0 | 243.6 | 135.2 | 44.6 | 44.9 | 1824.4 |
| Endocrine | 2.3 | 2.6 | 7.0 | 6.5 | 18.1 | 30.5 | 42.1 | 49.6 | 67.1 | 82.7 | 117.9 | 153.8 | 194.0 | 214.5 | 182.2 | 134.2 | 67.6 | 39.6 | 1412.3 |
| Cardiovascular | 6.4 | 2.0 | 1.6 | 6.7 | 10.9 | 21.0 | 35.7 | 57.1 | 99.0 | 166.3 | 237.3 | 288.6 | 355.8 | 390.4 | 313.6 | 231.4 | 141.6 | 108.1 | 2473.7 |
| Mental | 71.0 | 299.9 | 366.3 | 539.6 | 795.3 | 1299.9 | 1333.8 | 1149.6 | 1069.2 | 801.3 | 742.7 | 377.9 | 293.9 | 242.7 | 129.3 | 70.5 | 26.3 | 15.6 | 9624.7 |
| Neurological | 65.7 | 53.9 | 52.1 | 58.4 | 79.9 | 93.4 | 94.5 | 90.7 | 91.0 | 103.8 | 109.8 | 114.6 | 107.0 | 98.1 | 124.8 | 168.8 | 138.9 | 134.3 | 1779.8 |
| Hearing | 14.6 | 61.0 | 60.8 | 55.3 | 58.5 | 60.4 | 54.8 | 48.6 | 50.4 | 49.7 | 47.2 | 230.6 | 177.2 | 116.2 | 75.4 | 49.6 | 34.5 | 40.6 | 1285.6 |
| Respiratory | 74.2 | 173.0 | 154.6 | 127.9 | 156.7 | 222.3 | 229.0 | 194.7 | 192.9 | 180.5 | 188.6 | 194.1 | 273.5 | 288.3 | 226.6 | 130.8 | 56.4 | 56.3 | 3120.2 |
| Gastrointestinal | 9.1 | 9.7 | 12.2 | 24.2 | 61.9 | 94.7 | 97.1 | 102.1 | 89.8 | 99.4 | 108.6 | 96.0 | 87.3 | 79.1 | 49.7 | 28.3 | 13.0 | 9.7 | 1071.8 |
| Kidney | 0.0 | 0.0 | 0.0 | 4.0 | 5.8 | 24.7 | 27.4 | 30.2 | 61.1 | 77.2 | 144.8 | 183.2 | 236.5 | 333.3 | 306.6 | 164.5 | 107.7 | 13.7 | 1720.8 |
| Reproductive | 0.6 | 0.7 | 0.6 | 0.9 | 4.0 | 8.8 | 12.4 | 10.2 | 6.7 | 8.1 | 7.5 | 7.6 | 6.2 | 4.2 | 2.7 | 1.3 | 0.6 | 0.4 | 83.4 |
| Skin | 46.1 | 46.1 | 95.0 | 156.1 | 93.5 | 133.3 | 135.4 | 111.7 | 105.0 | 102.3 | 102.1 | 94.2 | 80.4 | 61.4 | 37.6 | 22.3 | 10.5 | 8.4 | 1441.6 |
| Musculoskeletal | 18.6 | 35.5 | 71.1 | 132.0 | 288.7 | 539.9 | 667.4 | 619.4 | 706.9 | 812.7 | 992.1 | 1052.4 | 1072.6 | 897.1 | 583.0 | 326.4 | 135.2 | 81.2 | 9031.9 |
| Oral | 31.4 | 38.3 | 26.3 | 28.2 | 46.7 | 56.5 | 48.5 | 49.7 | 46.9 | 43.9 | 47.6 | 98.9 | 91.0 | 74.6 | 46.3 | 45.0 | 19.3 | 12.9 | 852.0 |
| Blood | 23.4 | 2.6 | 3.8 | 3.4 | 4.9 | 6.8 | 7.9 | 7.0 | 6.0 | 6.7 | 8.8 | 10.0 | 12.7 | 11.4 | 11.8 | 12.1 | 9.4 | 13.5 | 162.1 |
| Intentional inj | 176.4 | 108.7 | 175.4 | 574.2 | 762.6 | 540.8 | 467.2 | 373.0 | 364.1 | 480.9 | 426.2 | 246.7 | 221.0 | 316.5 | 227.8 | 9.7 | 6.2 | 5.9 | 5483.5 |
| Unintentional inj | 102.0 | 61.4 | 89.5 | 195.4 | 255.8 | 263.1 | 265.8 | 247.0 | 243.1 | 283.5 | 276.1 | 190.4 | 163.4 | 148.7 | 104.9 | 7.3 | 3.9 | 1.4 | 2902.6 |
| Total | 1025.8 | 960.7 | 1180.9 | 1963.3 | 2754.7 | 3601.0 | 3758.4 | 3294.8 | 3389.8 | 3503.9 | 3817.2 | 3662.4 | 3743.9 | 3703.2 | 2697.3 | 1564.0 | 834.4 | 612.6 | 46068.3 |
| Non-Aboriginal Female | 0 | 5 | 10 | 15 | 20 | 25 | 30 | 35 | 40 | 45 | 50 | 55 | 60 | 65 | 70 | 75 | 80 | 85 |  |
| Infectious | 204.3 | 34.5 | 26.9 | 30.9 | 66.7 | 87.6 | 62.7 | 61.3 | 57.2 | 33.9 | 28.9 | 26.6 | 21.9 | 22.1 | 22.5 | 18.2 | 26.6 | 34.5 | 867.5 |
| Infant | 140.0 | 14.8 | 14.1 | 12.0 | 16.8 | 27.4 | 25.6 | 19.9 | 17.7 | 12.6 | 11.6 | 10.7 | 7.4 | 5.3 | 3.1 | 1.8 | 1.1 | 1.3 | 343.2 |
| Cancer | 4.1 | 3.7 | 1.2 | 6.9 | 6.2 | 33.9 | 63.9 | 71.5 | 88.1 | 149.2 | 154.2 | 158.2 | 174.4 | 145.4 | 108.0 | 78.7 | 40.4 | 37.7 | 1325.8 |
| Endocrine | 6.3 | 2.8 | 5.0 | 11.1 | 16.8 | 29.6 | 33.9 | 41.1 | 56.7 | 65.5 | 85.1 | 103.8 | 113.3 | 104.4 | 89.0 | 69.5 | 47.4 | 41.2 | 922.3 |
| Cardiovascular | 9.1 | 2.0 | 1.1 | 3.1 | 5.5 | 9.8 | 18.3 | 29.5 | 44.0 | 58.2 | 70.7 | 79.0 | 92.0 | 113.1 | 115.8 | 121.1 | 112.6 | 144.4 | 1029.4 |
| Mental | 34.5 | 142.2 | 241.2 | 404.2 | 653.8 | 995.3 | 990.9 | 848.8 | 727.9 | 599.6 | 516.2 | 352.5 | 251.1 | 120.6 | 85.6 | 35.7 | 21.5 | 13.6 | 7035.3 |
| Neurological | 95.7 | 58.2 | 56.2 | 76.4 | 124.1 | 163.0 | 169.0 | 144.3 | 152.2 | 157.4 | 135.6 | 117.0 | 102.1 | 91.6 | 95.0 | 125.0 | 109.3 | 188.4 | 2160.7 |
| Hearing | 11.4 | 55.8 | 52.9 | 49.9 | 53.0 | 57.4 | 53.4 | 48.0 | 50.3 | 51.8 | 48.6 | 59.7 | 198.7 | 132.4 | 85.6 | 59.0 | 48.9 | 61.2 | 1178.1 |
| Respiratory | 52.8 | 121.0 | 115.3 | 112.7 | 177.4 | 289.0 | 266.8 | 214.7 | 203.9 | 217.9 | 261.3 | 273.1 | 284.5 | 229.9 | 196.6 | 140.0 | 84.9 | 106.0 | 3347.7 |
| Gastrointestinal | 9.4 | 9.8 | 11.7 | 22.1 | 56.6 | 93.7 | 96.5 | 97.9 | 79.2 | 87.7 | 93.1 | 82.8 | 73.5 | 62.1 | 41.8 | 25.9 | 15.1 | 15.2 | 974.0 |
| Kidney | 0.1 | 0.0 | 0.0 | 4.5 | 2.8 | 22.2 | 29.1 | 27.0 | 18.8 | 38.7 | 69.1 | 70.1 | 63.8 | 89.9 | 130.4 | 91.6 | 57.3 | 0.2 | 715.5 |
| Reproductive | 0.7 | 0.4 | 14.6 | 66.9 | 169.4 | 320.9 | 328.7 | 206.9 | 117.0 | 101.9 | 75.9 | 106.2 | 80.6 | 58.0 | 36.0 | 18.8 | 9.9 | 8.6 | 1721.5 |
| Skin | 42.7 | 45.6 | 116.2 | 133.2 | 85.0 | 135.7 | 139.1 | 111.5 | 96.6 | 89.6 | 84.7 | 77.0 | 60.0 | 41.4 | 24.9 | 15.1 | 8.9 | 10.0 | 1317.2 |
| Musculoskeletal | 19.0 | 32.4 | 71.0 | 111.9 | 224.3 | 465.3 | 599.3 | 589.8 | 649.1 | 779.6 | 953.1 | 1079.3 | 1003.2 | 767.9 | 518.0 | 315.1 | 160.5 | 128.1 | 8466.9 |
| Oral | 28.6 | 23.7 | 18.7 | 24.8 | 37.9 | 39.0 | 26.6 | 36.2 | 34.8 | 36.3 | 31.8 | 82.6 | 66.8 | 50.5 | 31.8 | 34.6 | 19.4 | 16.3 | 640.3 |
| Blood | 38.2 | 7.5 | 14.7 | 27.1 | 28.0 | 36.0 | 41.6 | 72.3 | 73.6 | 59.3 | 36.0 | 26.3 | 27.7 | 20.9 | 13.5 | 11.7 | 12.0 | 24.3 | 570.4 |
| Intentional inj | 137.2 | 77.2 | 110.9 | 249.5 | 291.0 | 198.8 | 162.2 | 120.8 | 109.5 | 118.9 | 105.4 | 69.8 | 73.9 | 155.4 | 111.2 | 6.1 | 7.1 | 10.1 | 2114.9 |
| Unintentional inj | 75.8 | 80.7 | 89.0 | 80.9 | 92.0 | 95.8 | 88.1 | 69.8 | 65.6 | 66.5 | 60.8 | 50.0 | 58.6 | 118.7 | 84.6 | 3.1 | 2.4 | 1.7 | 1184.3 |
| Total | 909.9 | 712.1 | 960.7 | 1428.4 | 2107.6 | 3100.3 | 3195.7 | 2811.3 | 2642.3 | 2724.2 | 2822.1 | 2824.6 | 2753.5 | 2329.8 | 1793.4 | 1171.1 | 785.1 | 842.9 | 35914.8 |

Note: inj=injury

**(4) Cause decomposition of healthy life expectancy**

**Table A2. Decomposition of difference (contribution %) [rank] in healthy life expectancy at birth between Indigenous and non-Indigenous by cause category, Northern Territory of Australia, 2014-2018**

|  |  | Male |  | Female |
| --- | --- | --- | --- | --- |
| Infectious |  | 0.44(2%)[11] |  | 0.98(3%)[10] |
| Infant/Congenital | | -0.82(-3%)[17] |  | -0.87(-3%)[17] |
| Cancer |  | -0.88(-3%)[18] |  | -0.72(-2%)[15] |
| Endocrine |  | 7.85(30%)[1] |  | 10.36(31%)[1] |
| Cardiovascular | | 4.68(18%)[3] |  | 7.05(21%)[2] |
| Mental/Substance | | 0.9(3%)[8] |  | -0.7(-2%)[14] |
| Neurological | | 1.82(7%)[6] |  | 1.72(5%)[7] |
| Hearing/Vision | | 6.58(25%)[2] |  | 5.19(16%)[3] |
| Respiratory | | -0.81(-3%)[16] |  | 1.62(5%)[8] |
| Gastrointestinal | | -0.67(-3%)[15] |  | -0.67(-2%)[13] |
| Kidney/Urinary | | 2.77(10%)[4] |  | 4.16(12%)[4] |
| Reprod./Maternal | | -0.04(0%)[13] |  | -1.12(-3%)[18] |
| Skin |  | 0.45(2%)[10] |  | 0.63(2%)[11] |
| Musculoskeletal | | 0.56(2%)[9] |  | 2.43(7%)[6] |
| Oral |  | 1.62(6%)[7] |  | 1.42(4%)[9] |
| Blood/Metabolic | | 1.89(7%)[5] |  | 2.91(9%)[5] |
| Intentional inj. | | -0.06(0%)[14] |  | -0.23(-1%)[12] |
| Unintentional inj. | | 0.23(1%)[12] |  | -0.72(-2%)[16] |
| Total |  | 26.52(100%) |  | 33.45(100%) |

Note: inj=injury

**References**

1. Arriaga EE. Measuring and explaining the change in life expectancies. Demography*.* 1984;21:83-96.

2. Andreev EM, Shkolnikov VM, Begun AZ. Algorithm for decomposition of differences between aggregate demographic measures and its application to life expectancies, healthy life expectancies, parity-progression ratios and total fertility rates. Dem Res*.* 2002;7:499-522.
